# Risk factors for syphilis among newly admitted prisoners: A case–control study

**DOI:** 10.1371/journal.pone.0360956

**Published:** 2026-10-16

**Authors:** Thanaset Hiranbandit, Wutthichai Jariya

**Affiliations:** 1 Department of Community Health, Faculty of Public Health, Naresuan University, Phitsanulok, Thailand; 2 Office of Disease Prevention and Control Region 2, Department of Disease Control, Ministry of Public Health, Phitsanulok, Thailand; Tarbiat Modares University Faculty of Medical Sciences, IRAN, ISLAMIC REPUBLIC OF

## Abstract

**Background:**

Syphilis remains a major global public health concern in various groups, including people in prisons, where the burden is substantial. This study aimed to identify factors associated with syphilis infection among newly admitted prisoners in a province of Thailand.

**Methods:**

A case–control study was conducted among newly admitted prisoners in three correctional facilities. Cases and controls were randomly selected from newly admitted prisoners who had undergone syphilis screening at prison entry between 01/04/2025 and 30/09/2025. Data were collected using validated data collection instruments, including case record forms and structured questionnaires. Multivariable logistic regression analysis was used to identify factors associated with syphilis infection among newly admitted prisoners, with statistical significance set at *p*-value < 0.05.

**Results:**

A total of 51 cases and 204 controls were included in the study. In multivariable logistic regression analysis, homosexual or bisexual orientation (adjusted odds ratio [AOR] = 9.59; 95% confidence interval [CI]: 1.81–50.92), a history of sexually transmitted infections (AOR = 38.03; 95% CI: 10.46–138.31), anal intercourse (AOR = 4.47; 95% CI: 1.05–19.12), sexual intercourse without condom use (AOR = 13.82; 95% CI: 3.38–56.43), and injection drug use (AOR = 45.60; 95% CI: 2.88–722.12) were significantly associated with syphilis infection among newly admitted prisoners.

**Conclusions:**

Prison entry represents a critical opportunity for syphilis screening. Risk-based screening strategies targeting individuals with high-risk sexual behaviors and substance use should be prioritized to reduce syphilis transmission in correctional facilities.

## Introduction

Sexually transmitted infections (STIs) comprise a diverse group of bacterial, viral, and parasitic infections [1,2]. STIs are transmitted primarily through sexual contact, including vaginal, anal, and oral sex, while some infections may also be transmitted through blood exposure or vertical transmission [2,3]. Transmission is also associated with sexual risk behaviors, such as condomless sex and multiple sexual partners, while many STIs may remain asymptomatic and undetected [3]. The most common STIs include syphilis, gonorrhea, chlamydia, trichomoniasis, genital herpes, human papillomavirus infection, and HIV infection [2,4]. Among these STIs, syphilis remains a major public health concern worldwide [5].

Syphilis, a chronic progressive infection caused by *Treponema pallidum*, is primarily transmitted through sexual contact, blood exposure, and vertical transmission [3,6]. The infection causes significant health complications, such as nervous system damage, vision problems, and hearing loss. It is also associated with an increased risk of HIV infection [7,8]. Asymptomatic infection, high-risk behaviors, limited access to screening, and delayed treatment are associated with ongoing syphilis transmission [9]. Diagnosis and management remain challenging due to diverse clinical manifestations and complex serological test results [10]. These challenges underscore the need for effective syphilis prevention and control [11,12].

The number of new syphilis infections worldwide is increasing, rising from 7.1 million among adults aged 15–49 in 2020 to 8.0 million in 2022 [12]. The World Health Organization has set a target to reduce annual syphilis infections tenfold by 2030 [12]. To achieve this goal, effective strategies are needed to reduce syphilis among high-burden populations, particularly in prisons, where the burden is substantial [5,13].

A systematic review and meta-analysis found considerable global prevalence of syphilis among incarcerated populations, with a pooled prevalence of 4.5% (95% CI: 3.6–5.6) among adults [13]. Prevalence was higher in low- and middle-income countries (6.9%, 95% CI: 5.2–9.0) than in high-income countries (2.6%, 95% CI: 2.0–3.5) [13]. Estimates varied widely at the country level; in Brazil, lifetime prevalence ranged from 10.5% to 11.6% [14,15].. By contrast, a low prevalence of 0.6% was reported in Iraq [16]. In Southeast Asia, syphilis prevalence among incarcerated populations was reported at 6.3–7.0% in Indonesia [17,18] and 4.8% among male prisoners in Thailand [19].

The high burden of syphilis in prison populations occurs in the context of behavioral and structural vulnerabilities [13,18,20]. Sexual risk behaviors, substance use, and limited access to prevention, screening, and treatment have been reported in correctional settings [18,20]. Overcrowding and restricted access to healthcare have also been reported as challenges for STI prevention and management in prisons [20].

The substantial burden of syphilis in correctional settings has important implications for STI control and prison health services [13]. Undiagnosed or inadequately treated syphilis remains a concern for ongoing transmission within correctional facilities [13,21]. This situation highlights the need for adequate screening, treatment, and follow-up services in prison health systems [21,22].

Syphilis in prison populations is associated with several individual and behavioral factors, including age [15–17], educational attainment [14], income level [14], sexual orientation [15], a history of STIs [23], HIV-positive status [15,24], and high-risk sexual behaviors [24]. Individuals with these risk factors may enter correctional facilities with undiagnosed syphilis and potentially introduce the infection upon entry [25].

Screening at prison entry provides an opportunity to detect syphilis infections acquired before incarceration [3,12,18]. Limited access to screening and treatment in correctional settings is associated with ongoing STI transmission, particularly when infections remain undetected [20,22,25]. Therefore, timely screening and treatment of newly admitted prisoners are important components of syphilis prevention and control in prisons [18,21,26].

Despite the substantial burden of syphilis in prisoners [13], existing studies examining associated factors have primarily used cross-sectional designs and have focused on general incarcerated populations rather than newly admitted prisoners [14–17,23,24]. Few studies have specifically examined factors associated with syphilis among prisoners at admission [27]. Therefore, this study aimed to identify factors associated with syphilis among newly admitted prisoners in Thailand using a case-control design. The findings are expected to inform targeted screening and early intervention strategies to reduce syphilis transmission within correctional facilities.

## Materials and methods

### Study design, setting, and period

A case–control study was conducted to identify factors associated with syphilis infection among newly admitted prisoners. This design was selected because case–control studies are efficient for examining factors associated with relatively uncommon outcomes [28]. Although routine syphilis screening results were available for all newly admitted prisoners, information on the personal and behavioral factors examined in this study was not routinely recorded and required direct collection from participants. The case–control design therefore allowed comparison of these factors between prisoners with syphilis and those without syphilis, while avoiding administration of a sensitive questionnaire to all screened prisoners.

The study was conducted in three prisons in a province in Health Region 2 (lower northern Thailand). The province was selected as the study setting due to its high burden of syphilis among newly admitted prisoners. Surveillance data from the Department of Disease Control [29] indicated that, in 2024, the prevalence of syphilis among newly admitted prisoners in Thailand was 2.40%, while a higher prevalence was observed in Health Region 2 (2.86%). Within this region, the selected province reported the highest prevalence (4.48%), exceeding both regional and national levels. The name of the province is not disclosed to protect the confidentiality of the study setting and to minimize potential stigma associated with reporting a high prevalence of syphilis among prisoners.

Participants were formally recruited and enrolled into the study from 01/10/2025 to 26/12/2025. The sampling frame consisted of newly admitted prisoners who had undergone syphilis screening at prison entry between 01/04/2025 and 30/09/2025. This screening period was used to identify eligible cases and controls and was not the recruitment period. Data were collected from the selected cases and controls during the recruitment period (01/10/2025 to 26/12/2025). This study was reported in accordance with the Strengthening the Reporting of Observational Studies in Epidemiology (STROBE) guidelines (S1 File) [30].

### Study population and sample

The study population consisted of newly admitted prisoners who had been incarcerated for ≤1 month prior to assignment to residential units and had undergone syphilis screening. Syphilis status was ascertained from routine prison intake screening and medical records in accordance with national guidelines [26,31]. All eligible prisoners remained incarcerated at the time of data collection.

Cases were defined as prisoners who tested positive for syphilis using the reverse screening algorithm and received a clinical diagnosis of syphilis by a physician. Diagnoses were recorded using ICD-10 codes A51.x for early syphilis, A52.x for late syphilis, or A53.x for other and unspecified syphilis [32]. Controls were defined as prisoners screened using the same algorithm who did not receive a clinical diagnosis of syphilis.

The sample size was based on a previous study [23] that identified a history of STIs within the past 12 months as a significant risk factor for syphilis, with exposure proportions of 0.20 among cases and 0.057 among controls. OpenEpi (Version 3.01) was used to calculate the sample size for an unmatched case–control study [33]. The calculation was based on the Fleiss method [34], with the following assumptions: a two-sided 95% confidence level, 80% power, a control-to-case ratio of 4:1, an exposure proportion of 5.7% among controls, and an expected odds ratio of 4.14. The minimum required sample size was estimated to be 46 cases and 184 controls. To account for potential incomplete data, the number of cases was increased by 10%, resulting in a final sample of 51 cases and 204 controls.

Participants were selected from the sampling frame of newly admitted prisoners who had undergone syphilis screening at prison entry between 01/04/2025 and 30/09/2025 (N = 1,818). After excluding 323 individuals due to release or transfer, 1,495 participants were eligible, including 71 cases and 1,424 controls. Participants were stratified by prison (Prison A, Prison B, and Prison C); cases were selected using simple random sampling, and controls were randomly selected within each prison at a 4:1 ratio until the required sample size was reached (Fig 1). The inclusion criteria were: (1) aged 18 years or older; (2) having undergone syphilis screening at prison entry between 01/04/2025 and 30/09/2025; (3) ability to communicate in Thai; and (4) willingness to participate. The exclusion criteria were: (1) release from prison before data collection and (2) transfer to other prisons.

**Fig 1:**
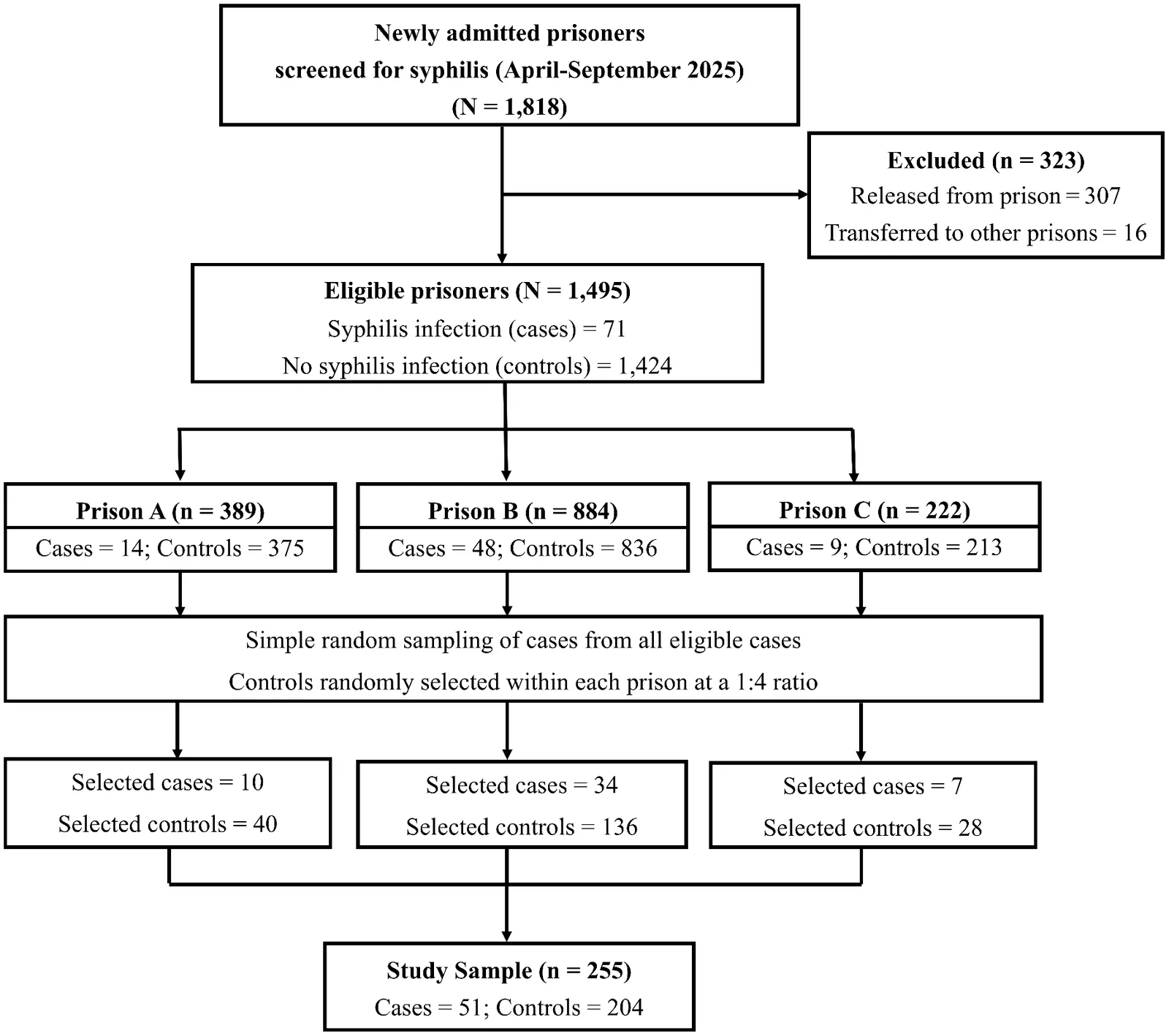
Flow diagram of participant selection for the case–control study of syphilis infection among newly admitted prisoners.

### Data collection instruments and laboratory tests

#### Questionnaire and case record form.

The questionnaire (S2 File) consisted of two sections. The first section assessed personal factors with eight closed-ended questions: biological sex, age, educational attainment, occupation, marital status, gender identity, sexual orientation, and a history of STIs within the past 12 months prior to incarceration. Age was categorized into three groups (18–24, 25–44, and ≥45 years) based on classifications from the National Statistical Office of Thailand [35].

The second section assessed behavioral factors with eleven closed-ended questions: sexual intercourse, vaginal intercourse, anal intercourse, oral sex, buying or selling sexual services, sexual intercourse without condom use, injection drug use, non-injection drug use, substance use during sexual intercourse, tattooing or body piercing, and sharing personal items. All 11 behavioral variables were based on self-reported experiences within the past 12 months prior to incarceration. Vaginal intercourse, anal intercourse, and oral sex were assessed as separate, non-mutually exclusive variables; therefore, participants could report more than one type of sexual intercourse.

The case record form (S3 File) was used to extract data on one independent variable related to personal factor (HIV infection status at prison entry) and the dependent variable (syphilis infection status at prison entry), based on prison screening records.

The content validity of the questionnaire and case record form was evaluated by a panel of seven experts. The item-level content validity index (I-CVI) was 1.00 for all items. The scale-level content validity indices, including the average method (S-CVI/Ave) and the universal agreement method (S-CVI/UA), were both 1.00, indicating that the instrument met the criteria for content validity [36]. A pilot test of the questionnaire and case record form was conducted among 30 newly admitted prisoners in another prison in a different province with similar characteristics to the study setting. The pilot test indicated that the questionnaire was generally well understood, with no missing responses. Nevertheless, minor modifications were made to improve clarity. Because the questionnaire consisted of distinct factual and behavioral items rather than multiple items measuring a common latent construct, internal consistency reliability (e.g., Cronbach’s alpha) was not applicable. The pilot test was therefore used primarily to assess the clarity, comprehensibility, and feasibility of the questionnaire.

#### Laboratory tests.

Venous blood samples were collected by registered nurses working at prison health facilities from newly admitted prisoners. Blood specimens were transported to the central laboratory of the referral hospital. The collected samples were used for screening of syphilis infection in accordance with national guidelines [26,31].

Syphilis testing followed a reverse screening algorithm [31]. Initial screening was performed using a rapid treponemal diagnostic test (RDT). Samples with reactive results were subsequently tested using the Rapid Plasma Reagin (RPR) test, with titers recorded for quantitative assessment. In cases of discordant results between treponemal and non-treponemal tests (e.g., reactive RDT with non-reactive RPR), the *Treponema pallidum* particle agglutination (TPPA) assay was used for confirmation.

### Data collection procedures

In accordance with prison regulations and security measures, the researchers obtained a list of newly admitted prisoners from prison health records and identified cases and controls based on predefined criteria and a random sampling procedure. Following completion of consent procedures, the researchers extracted clinical and laboratory data, including syphilis screening results at prison entry by using a case record form. The researchers then approached each selected participant individually and asked them to complete a questionnaire. Before participants completed the questionnaire, the researchers explained that the behavioral questions referred specifically to experiences during the 12 months before the current incarceration and asked participants to answer based on their behaviors before entering prison.

To minimize social desirability bias on sensitive topics such as sexual behavior and drug use, participants completed the questionnaire independently in a private room without the presence of prison officers or researchers. The researchers remained available outside the room to provide clarification on questionnaire items or assist participants who had difficulty reading or understanding the questions upon request.

### Ethical considerations

This study was approved by the Human Research Ethics Committee of Naresuan University on 03/09/2025 (IRB No. P2-0254/2025; COA No. 310/2025). The research conformed to the ethical principles of the Declaration of Helsinki. Participants were assured that participation in the study would not result in any legal, medical, or social penalties. They were also informed of their right to refuse participation or withdraw from the study at any time without consequences.

Participation was initiated only after informed consent was obtained from all participants. The researchers conducted the data collection themselves in accordance with prison regulations and security measures. Correctional officers were involved only in facilitating access to the study areas and calling selected prisoners based on the sampling codes. They were not involved in recruiting participants, providing study information, distributing questionnaires, or obtaining informed consent. All study information and consent procedures were provided directly by the researchers.

All personal identifiers were removed, prison sites were anonymized using alphabetical codes, and participants were assigned unique numeric codes. Data were securely stored and findings reported in aggregate form. Per standard prison health protocols, participants who tested positive for syphilis received treatment at the prison or were referred to a hospital for treatment.

### Data management and analysis

Data (S4 Data) were coded and entered into a computer database and analyzed using jamovi version 2.3.28 [37] for descriptive, bivariate, and multivariable logistic regression analyses, while R version 4.5.3 [38] was used for Firth penalized logistic regression in the sensitivity analysis. Descriptive statistics, including frequencies and percentages, were used to describe participants’ characteristics according to personal and behavioral factors. Categorical independent variables were compared between cases and controls using Chi-square test when expected cell counts were adequate; otherwise, Fisher’s exact test was applied.

Factors associated with syphilis infection among newly admitted prisoners were examined using univariable and multivariable logistic regression analyses. Univariable analyses were performed using simple logistic regression, with results presented as crude odds ratios (CORs) and 95% confidence intervals (CIs). Variables with *p*-values < 0.25 were included in the multivariable model [39]. Prior to multivariable analysis, multicollinearity was assessed using variance inflation factors (VIFs) and tolerance [40]. All variables showed VIF values < 5 and tolerance > 0.2, indicating no significant multicollinearity. Multivariable logistic regression was then performed using the backward Wald variable selection method, with results reported as adjusted odds ratios (AORs) and 95% CI. Statistical significance was set at *p*-value < 0.05. Nagelkerke’s pseudo-R^2^ was used to assess the explanatory power of the model [41]. In addition, because the injection drug use variable was rare and the multivariable logistic regression estimate was imprecise, a sensitivity analysis was conducted using Firth penalized logistic regression to reduce small-sample and sparse-data bias [42,43]. The same variables retained in the final multivariable logistic regression model were included in the Firth model, and results were reported as Firth-adjusted odds ratios with 95% CI.

## Results

### Personal factors of participants

Among the 255 newly admitted prisoners, the majority were male (76.5%) and aged 25–44 years (69.0%). Most participants were single (66.3%), and 66.7% had less than upper secondary education. The most common occupation was government or private-sector employment (59.2%). Most participants identified as cisgender (96.1%) and reported heterosexual orientation (89.8%). The majority also reported no history of STIs (88.6%), while only 1.2% of participants were HIV-positive (Table 1).

**Table 1:** Personal factors of participants. (n = 255).

Variables	Total (n = 255) n (%)	Cases (n = 51) n (%)	Controls (n = 204) n (%)	χ²	*p*-value
**Biological sex** ^**†**^					
Male	195 (76.5)	39 (76.5)	156 (76.5)	0.00	1.000
Female	60 (23.5)	12 (23.5)	48 (23.5)		
**Age (years)** ^**†**^					
18–24	34 (13.3)	11 (21.6)	23 (11.3)	3.59	0.166
25–44	176 (69.0)	33 (64.7)	143 (70.1)		
≥ 45	45 (17.6)	7 (13.7)	38 (18.6)		
**Educational attainment** ^**†**^					
Less than upper secondary education	169 (66.3)	36 (70.6)	133 (65.2)	0.49	0.482
Upper secondary education or higher	86 (33.7)	15 (29.4)	71 (34.8)		
**Occupation** ^**†**^					
Government or private-sector employee	151 (59.2)	30 (58.8)	121 (59.3)	1.08	0.582
Self-employed	87 (34.1)	16 (31.4)	71 (34.8)		
Unemployed	17 (6.7)	5 (9.8)	12 (5.9)		
**Marital status** ^**†**^					
Married	86 (33.7)	18 (35.3)	68 (33.3)	0.07	0.791
Single	169 (66.3)	33 (64.7)	136 (66.7)		
**Gender identity** **^‡^**					
Transgender	10 (3.9)	6 (11.8)	4 (2.0)	N/A	0.005*
Cisgender	245 (96.1)	45 (88.2)	200 (98.0)		
**Sexual orientation** ^**†**^					
Homosexual / bisexual	26 (10.2)	12 (23.5)	14 (6.9)	12.38	< 0.001*
Heterosexual only	229 (89.8)	39 (76.5)	190 (93.1)		
**HIV infection status** **^‡^**					
HIV-positive	3 (1.2)	3 (5.9)	0 (0.0)	N/A	0.008*
HIV-negative	252 (98.8)	48 (94.1)	204 (100.0)		
**History of STIs** ^**†**^					
Yes	29 (11.4)	25 (49.0)	4 (2.0)	89.64	< 0.001*
No	226 (88.6)	26 (51.0)	200 (98.0)		

† Chi-square test; ‡ Fisher’s exact test; *Significant at a *p*-value < 0.05.

Abbreviations: HIV, human immunodeficiency virus; STI, sexually transmitted infection.

Comparisons between cases and controls indicated that a higher proportion of cases reported homosexual or bisexual orientation than controls (p < 0.001). The proportion of transgender individuals was also higher among cases (p = 0.005). HIV-positive status was more common in cases (p = 0.008). A higher proportion of cases reported the history of STIs (p < 0.001). In contrast, no statistically significant differences were found between the two groups in terms of biological sex (p = 1.000), age group (p = 0.166), educational attainment (p = 0.482), occupation (p = 0.582), and marital status (p = 0.791) (Table 1).

### Behavioral factors of participants

Among the 255 newly admitted prisoners, the majority reported sexual intercourse (74.5%) and vaginal intercourse (71.0%), while 5.9% reported engaging in anal intercourse and 34.9% reported oral sex. Most participants reported no history of buying or selling sexual services (88.2%). Nearly half of the participants reported having sexual intercourse without condom use (57.3%). Non-injection drug use was reported by 60.8% of participants, while only 1.6% reported injection drug use. Additionally, 32.9% reported substance use during sexual intercourse, 30.6% reported tattooing or body piercing, and 60.4% reported sharing personal items (Table 2).

**Table 2:** Behavioral factors of participants. (n = 255).

Variables	Total (n = 255) n (%)	Cases (n = 51) n (%)	Controls (n = 204) n (%)	χ²	*p*-value
**Sexual intercourse** **^‡^**					
Yes	190 (74.5)	50 (98.0)	140 (68.6)	N/A	< 0.001*
No	65 (25.5)	1 (2.0)	64 (31.4)		
**Vaginal intercourse** **^†^**					
Yes	181 (71.0)	44 (86.3)	137 (67.2)	6.67	0.010*
No	74 (29.0)	7 (13.7)	67 (32.8)		
**Anal intercourse** **^‡^**					
Yes	15 (5.9)	10 (19.6)	5 (2.5)	N/A	< 0.001*
No	240 (94.1)	41 (80.4)	199 (97.5)		
**Oral sex** **^†^**					
Yes	89 (34.9)	27 (52.9)	62 (30.4)	9.01	0.003*
No	166 (65.1)	24 (47.1)	142 (69.6)		
**Buying or selling sexual services** **^†^**					
Yes	30 (11.8)	8 (15.7)	22 (10.8)	0.93	0.334
No	225 (88.2)	43 (84.3)	182 (89.2)		
**Sexual intercourse without condom use** **^†^**					
Yes	146 (57.3)	46 (90.2)	100 (49.0)	24.88	< 0.001*
No	109 (42.7)	5 (9.8)	104 (51.0)		
**Injection drug use** **^‡^**					
Yes	4 (1.6)	3 (5.9)	1 (0.5)	N/A	0.029*
No	251 (98.4)	48 (94.1)	203 (99.5)		
**Non-injection drug use** **^†^**					
Yes	155 (60.8)	35 (68.6)	120 (58.8)	1.62	0.202
No	100 (39.2)	16 (31.4)	84 (41.2)		
**Substance use during sexual intercourse** **^†^**					
Yes	84 (32.9)	27 (52.9)	57 (27.9)	12.50	< 0.001*
No	171 (67.1)	24 (47.1)	147 (72.1)		
**Tattooing or body piercing** **^†^**					
Yes	78 (30.6)	27 (52.9)	51 (25.0)	14.42	< 0.001*
No	177 (69.4)	24 (47.1)	153 (75.0)		
**Sharing personal items** **^†^**					
Yes	154 (60.4)	32 (62.7)	122 (59.8)	0.15	0.701
No	101 (39.6)	19 (37.3)	82 (40.2)		

† Chi-square test; ‡ Fisher’s exact test; *Significant at a *p*-value < 0.05.

Comparisons between cases and controls indicated that a higher proportion of cases reported having sexual intercourse than controls (p < 0.001). Histories of vaginal intercourse (p = 0.010), anal intercourse (p < 0.001), and oral sex (p = 0.003) were more common among cases. A higher proportion of cases reported sexual intercourse without condom use (p < 0.001), substance use during sexual intercourse (p < 0.001), tattooing or body piercing (p < 0.001), and injection drug use (p = 0.029). In contrast, no statistically significant differences were observed between the two groups in terms of buying or selling sexual services (p = 0.334), non-injection drug use (p = 0.202), or sharing personal items (p = 0.701) (Table 2).

### Personal and behavioral factors associated with syphilis infection

Table 3 shows that three personal factors were significantly associated with syphilis infection among newly admitted prisoners. Transgender participants had significantly higher odds of syphilis infection compared with cisgender participants (COR = 6.67; 95% CI: 1.81–24.61). Participants reporting homosexual or bisexual orientation had higher odds of syphilis infection than those reporting heterosexual orientation (COR = 4.18; 95% CI: 1.80–9.72). Participants with a history of STIs had higher odds of syphilis infection than those without (COR = 48.08; 95% CI: 15.50–149.10).

**Table 3:** Univariable logistic regression analysis of personal and behavioral factors associated with syphilis infection among newly admitted prisoners. (n = 255).

Variables	Cases (n = 51) n (%)	Controls (n = 204) n (%)	Crude OR (95% CI)
***Personal factors***			
**Biological sex**			
Male	39 (76.5)	156 (76.5)	1.00 (0.49–2.06)
Female	12 (23.5)	48 (23.5)	Ref.
**Age (years)**			
18-24	11 (21.6)	23 (11.3)	2.60 (0.88–7.64)
25-44	33 (64.7)	143 (70.1)	1.25 (0.51–3.05)
≥ 45	7 (13.7)	38 (18.6)	Ref.
**Educational attainment**			
Less than upper secondary education	36 (70.6)	133 (65.2)	1.28 (0.66–2.50)
Upper secondary education or higher	15 (29.4)	71 (34.8)	Ref.
**Occupation**			
Government or private-sector employee	30 (58.8)	121 (59.3)	1.10 (0.56–2.16)
Unemployed	5 (9.8)	12 (5.9)	1.85 (0.57–5.99)
Self-employed	16 (31.4)	71 (34.8)	Ref.
**Marital status**			
Married	18 (35.3)	68 (33.3)	1.09 (0.57–2.08)
Single	33 (64.7)	136 (66.7)	Ref.
**Gender identity**			
Transgender	6 (11.8)	4 (2.0)	6.67 (1.81–24.61)
Cisgender	45 (88.2)	200 (98.0)	Ref.
**Sexual orientation**			
Homosexual / bisexual	12 (23.5)	14 (6.9)	4.18 (1.80–9.72)
Heterosexual only	39 (76.5)	190 (93.1)	Ref.
**HIV infection status^†^**			
HIV-positive	3 (5.9)	0 (0.0)	2.45x10^7^ (0.00–Inf)
HIV-negative	48 (94.1)	204 (100.0)	Ref.
**History of STIs**			
Yes	25 (49.0)	4 (2.0)	48.08 (15.50–149.10)
No	26 (51.0)	200 (98.0)	Ref.
***Behavioral factors***			
**Sexual intercourse**			
Yes	50 (98.0)	140 (68.6)	22.86 (3.09–169.03)
No	1 (2.0)	64 (31.4)	Ref.
**Vaginal intercourse**			
Yes	44 (86.3)	137 (67.2)	3.07 (1.31–7.19)
No	7 (13.7)	67 (32.8)	Ref.
**Anal intercourse**			
Yes	10 (19.6)	5 (2.5)	9.71 (3.15–29.90)
No	41 (80.4)	199 (97.5)	Ref.
**Oral sex**			
Yes	27 (52.9)	62 (30.4)	2.58 (1.38–4.82)
No	24 (47.1)	142 (69.6)	Ref.
**Buying or selling sexual services**			
Yes	8 (15.7)	22 (10.8)	1.54 (0.64–3.69)
No	43 (84.3)	182 (89.2)	Ref.
**Sexual intercourse without condom use**			
Yes	46 (90.2)	100 (49.0)	9.57 (3.65–25.06)
No	5 (9.8)	104 (51.0)	Ref.
**Injection drug use**			
Yes	3 (5.9)	1 (0.5)	12.69 (1.29–124.65)
No	48 (94.1)	203 (99.5)	Ref.
**Non-injection drug use**			
Yes	35 (68.6)	120 (58.8)	1.53 (0.80–2.95)
No	16 (31.4)	84 (41.2)	Ref.
**Substance use during sexual intercourse**			
Yes	27 (52.9)	57 (27.9)	2.90 (1.55–5.44)
No	24 (47.1)	147 (72.1)	Ref.
**Tattooing or body piercing**			
Yes	27 (52.9)	51 (25.0)	3.38 (1.79–6.37)
No	24 (47.1)	153 (75.0)	Ref.
**Sharing personal items**			
Yes	32 (62.7)	122 (59.8)	1.13 (0.60–2.13)
No	19 (37.3)	82 (40.2)	Ref.

† The crude OR and 95% CI for HIV infection status were unstable because no HIV-positive participants were observed in the control group, resulting in complete separation.

Abbreviations: CI, confidence interval; Inf, infinity; OR, odds ratio; Ref., reference category; HIV, human immunodeficiency virus; STI, sexually transmitted infection.

Table 3 presents that eight behavioral factors were significantly associated with syphilis infection among newly admitted prisoners. Participants who reported sexual intercourse had higher odds of syphilis infection compared with those who did not (COR = 22.86; 95% CI: 3.09–169.03). Histories of vaginal intercourse (COR = 3.07; 95% CI: 1.31–7.19), anal intercourse (COR = 9.71; 95% CI: 3.15–29.90), and oral sex (COR = 2.58; 95% CI: 1.38–4.82) were also associated with higher odds of syphilis infection. In addition, sexual intercourse without condom use (COR = 9.57; 95% CI: 3.65–25.06), injection drug use (COR = 12.69; 95% CI: 1.29–124.65), substance use during sexual intercourse (COR = 2.90; 95% CI: 1.55–5.44), and tattooing or body piercing (COR = 3.38; 95% CI: 1.79–6.37) were significantly associated with syphilis infection.

### Risk factors associated with syphilis infection

In the multiple logistic regression analysis (Table 4), two personal factors remained independently associated with syphilis infection. Participants reporting homosexual or bisexual orientation had higher odds of syphilis infection (AOR = 9.59; 95% CI: 1.81–50.92). A history of STIs was also associated with higher odds of infection (AOR = 38.03; 95% CI: 10.46–138.31). Three behavioral factors remained independently associated with syphilis infection. Anal intercourse (AOR = 4.47; 95% CI: 1.05–19.12), sexual intercourse without condom use (AOR = 13.82; 95% CI: 3.38–56.43), and injection drug use (AOR = 45.60; 95% CI: 2.88–722.12) were all associated with higher odds of syphilis infection. The final model was statistically significant overall (χ² = 111, df = 5, *p*-value < 0.001), with a Nagelkerke’s R² of 0.558.

**Table 4:** Multivariable logistic regression analysis of factors associated with syphilis infection among newly admitted prisoners. (n = 255).

Variables	Crude OR (95% CI)	Adjusted OR^†^ (95% CI)	Firth-adjusted OR^‡^ (95% CI)
***Personal factors***			
**Sexual orientation**			
Homosexual / bisexual	4.18 (1.80–9.72)	9.59 (1.81–50.92)	8.24 (1.76–42.63)
Heterosexual only	Ref.	Ref.	Ref.
**History of STIs**			
Yes	48.08 (15.50–149.10)	38.03 (10.46–138.31)	31.82 (10.28–123.09)
No	Ref.	Ref.	Ref.
***Behavioral factors***			
**Anal intercourse**			
Yes	9.71 (3.15–29.90)	4.47 (1.05–19.12)	4.32 (1.02–17.32)
No	Ref.	Ref.	Ref.
**Sexual intercourse without condom use**			
Yes	9.57 (3.65–25.06)	13.82 (3.38–56.43)	10.44 (3.32–43.91)
No	Ref.	Ref.	Ref.
**Injection drug use**			
Yes	12.69 (1.29–124.65)	45.60 (2.88–722.12)	30.33 (3.46–500.46)
No	Ref.	Ref.	Ref.

† Model fit: χ² = 111, df = 5, p-value < 0.001; Nagelkerke’s R² = 0.558

‡ Firth penalized logistic regression was performed as a sensitivity analysis to reduce potential sparse-data bias, particularly for injection drug use.

Abbreviations: AOR, adjusted odds ratio; CI, confidence interval; OR, odds ratio; Ref., reference category; STI, sexually transmitted infection.

Firth penalized logistic regression was performed as a sensitivity analysis to account for potential sparse-data bias. The results were broadly consistent with those of the conventional logistic regression. The association between injection drug use and syphilis remained strong, although the effect estimate was attenuated (AOR = 30.33, 95% CI 3.46–500.46), indicating considerable uncertainty due to the small number of participants reporting injection drug use (Table 4).

## Discussion

This case–control study aimed to identify factors associated with syphilis infection among newly admitted prisoners in Thailand. Newly admitted prisoners represent a critical opportunity for treatment and preventive interventions [44]. Screening at prison entry provides an opportunity to identify syphilis infections and facilitate timely treatment within correctional facilities [44]. The findings of this study identified two personal factors and three behavioral factors associated with syphilis infection among newly admitted prisoners.

Homosexual or bisexual orientation was associated with higher odds of syphilis infection. This finding is consistent with prior research demonstrating that homosexual preference was associated with active syphilis in male prisoners [15]. A global systematic review and meta-analysis reported that the prevalence of syphilis was substantially higher among men who have sex with men (MSM) than in heterosexual populations [45]. Previous studies suggested that individuals with homosexual orientation, such as MSM, are part of sexual networks with a higher baseline prevalence of syphilis [46,47]. Bisexual individuals were also described in prior studies as a potential ‘bridge population’ connecting higher-prevalence groups with lower-prevalence heterosexual networks [48,49].

A history of STIs was also associated with syphilis infection. This finding is in line with a previous study reporting a significantly higher prevalence of syphilis among incarcerated women with a history of STIs compared to those without a prior diagnosis [23]. Another study described prior STIs as a proxy for sexual risk behaviors, such as multiple sexual partners, unprotected sex, and substance use [50]. The CDC STI Treatment Guidelines also identify a history of STIs as an indicator of ongoing sexual risk behaviors [3]. Beyond behavioral risk, previous studies also described a history of STIs as being associated with greater susceptibility to subsequent infections, possibly through mucosal disruption that could facilitate pathogen entry into submucosal tissues [51–53].

Anal intercourse was identified as a factor associated with syphilis infection. This finding aligns with previous studies reporting that a higher frequency of anal intercourse was associated with syphilis infection [54]. A prior study described rectal mucosal inflammation and related mucosal changes among MSM engaging in receptive anal intercourse [55]. Condomless receptive anal intercourse was associated with markers of rectal mucosal inflammation [56], while lubrication practices during receptive anal sex were also examined in relation to STI risk [57]. Evidence from previous studies [55–57] provides biological and behavioral context for the association observed in this study, although these contextual factors were not directly assessed in the present study.

Sexual intercourse without condom use was associated with higher odds of syphilis infection. This finding is consistent with previous research showing that condomless sex was associated with syphilis infection [56]. Previous studies indicated that condomless sex can involve direct contact with syphilitic lesions, even when such lesions are small or invisible [3,10]. *Treponema pallidum* can be transmitted through microscopic abrasions in the skin or mucous membranes during sexual activity [58]. Although condoms reduce the risk of syphilis transmission, the CDC STI Treatment Guidelines note that protection is limited to areas covered by the condom, and transmission can still occur through contact with lesions in uncovered areas [3]. Condom provision remains a relevant component of STI prevention in correctional settings [59].

Injecting drug use was another factor associated with syphilis infection. This finding is consistent with a previous study indicating that the prevalence of syphilis among people who inject drugs is up to six times higher than that observed in the general population [60]. Another study reported that female prisoners who injected drugs during sexual activity had a higher risk of syphilis infection than those who did not [15]. Previous research also described injecting drug use as being associated with impaired judgment, reduced behavioral control, and high-risk sexual behaviors, including condomless sex, transactional sex for money or drugs, and multiple sexual partners [61].

The strengths of this study can be summarized in three main points. First, the case–control design was appropriate for identifying risk factors for a relatively uncommon outcome within a defined population. Second, using laboratory-confirmed syphilis diagnoses based on standard testing algorithms enhances the validity of outcome classification. Third, including both personal and behavioral variables provides a more comprehensive understanding of the risk factors for syphilis infection.

However, this study has several limitations. First, the case–control design prevents us from establishing causal relationships between the identified factors and syphilis infection. Second, because behavioral data were self-reported, they may be affected by recall or social desirability bias. Although participants were asked to report behaviors occurring before prison entry, the questionnaire was administered after syphilis screening had been completed; therefore, recall bias cannot be completely excluded. Third, the study was conducted among newly admitted prisoners in selected prisons, which may limit the generalizability of the findings to other settings. Fourth, prisoners who were released or transferred before data collection were excluded from the study. These individuals may have differed from those who remained incarcerated in sociodemographic characteristics, duration or circumstances of incarceration, or behavioral risk profiles. Their exclusion may therefore have introduced selection bias and may further limit the generalizability of the findings to all newly admitted prisoners. Because information on these characteristics was not available for comparison, the direction and magnitude of any resulting bias could not be determined. Fifth, although no problematic statistical multicollinearity was detected based on the VIF and tolerance values, some variables in the multivariable model were conceptually related. Anal intercourse and sexual intercourse without condom use were assessed as separate behaviors, but both may reflect similar sexual risk patterns. The history of STIs may also partly reflect previous sexual risk behaviors. Including these related variables in the same model may therefore have resulted in some overadjustment. Accordingly, the adjusted odds ratios, particularly the large estimate for a history of STIs, should be interpreted with caution. Finally, sparse data affected the precision of some estimates. Only four participants reported injection drug use, and only three participants were HIV-positive. For injection drug use, Firth penalized logistic regression produced a similar direction and magnitude of association, although the wide confidence interval indicated substantial uncertainty. For HIV status, all HIV-positive participants were in the syphilis case group, resulting in complete separation and an unstable crude odds ratio. These findings should therefore be interpreted with caution.

Future research should consider longitudinal study to better establish causal pathways of syphilis infection among prisoners. Intervention study should evaluate the effectiveness of targeted programs in reducing high-risk behaviors in prison settings. In addition, qualitative studies may provide in-depth understanding of the behavioral, social, and contextual factors contributing to syphilis infection among prisoners.

## Conclusion

This study identified several personal and behavioral factors independently associated with syphilis infection among newly admitted prisoners. Homosexual or bisexual orientation and the history of STIs were key personal factors associated with increased odds of infection. In addition, behavioral factors, including anal intercourse, sexual intercourse without condom use, and injection drug use, were significantly associated with syphilis infection.

These findings inform policy and practice by highlighting the importance of targeted screening at prison entry, particularly among individuals with high-risk sexual behaviors and substance use. Strengthening early detection, risk-based interventions, and linkage to appropriate treatment services may help reduce the burden of syphilis in prison settings.

## Supporting information

S1 File STROBE checklist for case–control study. (DOCX)

S2 File Structured questionnaire used for data collection. (DOCX)

S3 File Case record form used for data extraction. (DOCX)

S4 Data De-identified dataset and data dictionary. (XLSX)
