# Supplementary material for: Risk factors for syphilis among newly admitted prisoners: A case–control study
Source: PLoS One. 2026 Oct 16;21(10):e0360956. doi: 10.1371/journal.pone.0360956 (PMC13674709; doi:10.1371/journal.pone.0360956)
Supplement: Supplementary file 1 [file pone.0360956.s001.docx]

**STROBE Statement—checklist of items that should be included in reports of observational studies**

|  | Item No. | Recommendation | Page  No. | Relevant text from manuscript |
| --- | --- | --- | --- | --- |
| Title and abstract | 1 | (*a*) Indicate the study’s design with a commonly used term in the title or the abstract | 1 | Risk Factors for Syphilis among Newly Admitted Prisoners: A Case–Control Study |
|  |  | (*b*) Provide in the abstract an informative and balanced summary of what was done and what was found | 2 | The information regarding the methods and results are presented in the abstract |
| Introduction | | | |  |
| Background/rationale | 2 | Explain the scientific background and rationale for the investigation being reported | 3-4 | The scientific background and rationale are explained in the introduction section. |
| Objectives | 3 | State specific objectives, including any prespecified hypotheses | 4-5 | The objectives are stated in the last paragraph of the introduction section. |
| Methods | | | |  |
| Study design | 4 | Present key elements of study design early in the paper | 5 | The study design (case-control study) was presented in the section of study design, setting, and period |
| Setting | 5 | Describe the setting, locations, and relevant dates, including periods of recruitment, exposure, follow-up, and data collection | 5 | The setting, locations, and relevant dates, including periods of recruitment and data collection are described in the section of study design, setting, and period |
| Participants | 6 | a) ***Cohort study***—Give the eligibility criteria, and the sources and methods of selection of participants. Describe methods of follow-up b) ***Case-control study***—Give the eligibility criteria, and the sources and methods of case ascertainment and control selection. Give the rationale for the choice of cases and controls c) ***Cross-sectional study***—Give the eligibility criteria, and the sources and methods of selection of participants | 5-7 | According to the case-control study, the inclusion and exclusion criteria are explained in the section of study population and sample. The sources and methods of selection of participants are also described in the section of study population and sample. |
|  |  | d) ***Cohort study***—For matched studies, give matching criteria and number of exposed and unexposed e) ***Case-control study***—For matched studies, give matching criteria and the number of controls per case | 6-7 | No matching was performed between cases and controls. The number of controls per case (a control-to-case ratio of 4:1) is described in the section of study population and sample. |
| Variables | 7 | Clearly define all outcomes, exposures, predictors, potential confounders, and effect modifiers. Give diagnostic criteria, if applicable | 7-9 | The variables (risk factors and outcome) are defined and explained in the section of data collection instruments and laboratory tests. |
| Data sources/ measurement | 8* | For each variable of interest, give sources of data and details of methods of assessment (measurement). Describe comparability of assessment methods if there is more than one group | 7-9 | The variables (risk factors and outcome) are defined and explained in the section of data collection instruments and laboratory tests. |
| Bias | 9 | Describe any efforts to address potential sources of bias | 9, 23-24 | The effort to address potential sources of bias are described in the section of data collection procedures**.** Also, the potential biases were mentioned in the paragraph of limitations in the discussion section. |
| Study size | 10 | Explain how the study size was arrived at | 5-7 | The population and sample size are explained in the section of study population and sample. |

| Quantitative variables | | 11 | Explain how quantitative variables were handled in the analyses. If applicable, describe which groupings were chosen and why | 7-9 | Age was categorized into three groups (18–24, 25–44, and ≥45 years) based on classifications from the National Statistical Office of Thailand. All variables were analyzed as categorical variables as explained in the section of data collection instruments and laboratory tests. |  |  |  |
| --- | --- | --- | --- | --- | --- | --- | --- | --- |
| Statistical methods | | 12 | (*a*) Describe all statistical methods, including those used to control for confounding | 10-11 | Statistical methods are described in the section of data management and analysis. |  |  |  |
|  |  |  | (*b*) Describe any methods used to examine subgroups and interactions | - | Not applicable. |  |  |  |
|  |  |  | (*c*) Explain how missing data were addressed | - | Not applicable. |  |  |  |
|  |  |  | (*d*) *Cohort study*—If applicable, explain how loss to follow-up was addressed  *Case-control study*—If applicable, explain how matching of cases and controls was addressed  *Cross-sectional study*—If applicable, describe analytical methods taking account of sampling strategy | - | No matching was performed between cases and controls. |  |  |  |
|  |  |  | (*e*) Describe any sensitivity analyses | - | Not applicable. |  |  |  |
| Results | | | | | |  |  |  |
| Participants | | 13* | (a) Report numbers of individuals at each stage of study—eg numbers potentially eligible, examined for eligibility, confirmed eligible, included in the study, completing follow-up, and analysed | 5-7 | Numbers of individuals at each stage of study are reported in the section of study population and sample and Figure 1. |  |  |  |
|  |  |  | (b) Give reasons for non-participation at each stage | 5-7 | The reasons for non-participation at each stage are provided in the section of study population and sample and Figure 1. |  |  |  |
|  |  |  | (c) Consider use of a flow diagram | 7 | Figure 1 |  |  |  |
| Descriptive data | | 14* | (a) Give characteristics of study participants (eg demographic, clinical, social) and information on exposures and potential confounders | 12-15 | Table 1 & Table 2 |  |  |  |
|  |  |  | (b) Indicate number of participants with missing data for each variable of interest | - | Not applicable. |  |  |  |
|  |  |  | (c) *Cohort study*—Summarise follow-up time (eg, average and total amount) | - | Not applicable. |  |  |  |
| Outcome data | | 15* | *Cohort study*—Report numbers of outcome events or summary measures over time | - | Not applicable. |  |  |  |
|  |  |  | *Case-control study—*Report numbers in each exposure category, or summary measures of exposure | 12-18 | Table 1 - Table 3 |  |  |  |
|  |  |  | *Cross-sectional study—*Report numbers of outcome events or summary measures | - | Not applicable. |  |  |  |
| Main results | | 16 | (*a*) Give unadjusted estimates and, if applicable, confounder-adjusted estimates and their precision (eg, 95% confidence interval). Make clear which confounders were adjusted for and why they were included | 16-20 | Table 3 - Table 4 |  |  |  |
|  |  |  | (*b*) Report category boundaries when continuous variables were categorized | 7-9  12-18 | The report category boundaries, when continuous variables (age) were categorized, are explained in the section of data collection instruments and laboratory tests.  Table 1 and Table 3 |  |  |  |
|  |  |  | (*c*) If relevant, consider translating estimates of relative risk into absolute risk for a meaningful time period | - | Not applicable. |  |  |  |
| Other analyses | 17 | Report other analyses done—eg analyses of subgroups and interactions, and sensitivity analyses | | - | Not applicable. |  |  |  |
| Discussion | | | | | |  |  |  |
| Key results | 18 | Summarise key results with reference to study objectives | | 20-22 | Discussion section |  |  |  |
| Limitations | 19 | Discuss limitations of the study, taking into account sources of potential bias or imprecision. Discuss both direction and magnitude of any potential bias | | 23-24 | Discussion section (Limitations paragraph) |  |  |  |
| Interpretation | 20 | Give a cautious overall interpretation of results considering objectives, limitations, multiplicity of analyses, results from similar studies, and other relevant evidence | | 22-24 | Discussion section (strengths & Limitations paragraph) |  |  |  |
| Generalisability | 21 | Discuss the generalisability (external validity) of the study results | | 22-24 | Discussion section (strengths & Limitations paragraph) |  |  |  |
| Other information | |  | | | |  |  |  |
| Funding | 22 | Give the source of funding and the role of the funders for the present study and, if applicable, for the original study on which the present article is based | | - | Not applicable. |  |  |  |

***Give information separately for cases and controls in case-control studies and, if applicable, for exposed and unexposed groups in cohort and cross-sectional studies.**
