# Supplementary material for: Risk factors for syphilis among newly admitted prisoners: A case–control study
Source: PLoS One. 2026 Oct 16;21(10):e0360956. doi: 10.1371/journal.pone.0360956 (PMC13674709; doi:10.1371/journal.pone.0360956)
Supplement: Supplementary file 2 [file pone.0360956.s002.docx]

**Questionnaire on Personal and Behavioral Factors**

**Instructions:** Please place a check mark (√) in	box	that best reflects your actual situation for each item.

**Description of the questionnaire**

This questionnaire is used by researchers as a data collection tool for study participants. Respondents are asked to place a check mark (√) next to the response that most accurately reflects their personal circumstances and behaviors. The questionnaire was developed to examine personal and behavioral factors that may be associated with syphilis infection among newly admitted prisoners.

All information provided will be kept strictly confidential. Study findings will be reported only in aggregate form, and the data will be used solely for academic and research purposes.

The questionnaire consists of two sections, as follows:

**Section 1: Personal factors**

This section includes items on biological sex, age, educational attainment, occupation prior to incarceration, marital status, gender identity, sexual orientation, and history of sexually transmitted infections. A total of 8 items is included, and completion of this section takes approximately 15 minutes.

**Section 2: Behavioral factors**

This section includes items on history of sexual activity, sex of sexual partners, number
of sexual partners, history of vaginal intercourse, anal intercourse, and oral sex; history of buying or selling sexual services; history of sexual intercourse without condom use; history of injecting drug use; history of non-injecting drug use; history of substance use during sexual activity;
tattooing or body piercing; and sharing personal items with others. A total of 11 items is included, and completion of this section takes approximately 15 minutes.

**Instructions for Completing the Questionnaire**

Please read each question carefully and place a check mark (√) in the box next to
the response that most accurately reflects your actual situation. If a question includes space for additional information, please provide the details that best reflect the truth.

All information you provide will be kept strictly confidential and will be used solely
for research purposes. If any question causes discomfort or distress, you may choose to skip
that question without providing an answer.

**Definition of Response Options**

**1) Dichotomous response options (“Yes/No” or “Ever/Never”)**

- **Yes / Ever / Ever used** refers to having experienced the specified behavior or event at least once during the time period stated in the question.

- **No / Never / Never used** refers to having no experience with the specified behavior or event at any time.

**2) Open-ended response options**

For questions that require an open-ended response and provide a blank space
for additional information, participants are requested to write the relevant details in the space provided. Examples include specifying the name of a disease, the type of substance used,
or other information as requested in each item.

**Section 1: Personal Characteristics**

| **Item** | **Question** | **Response** |
| --- | --- | --- |
| 1 | Sex at birth | 1. Male |
|  |  | 2. Female |
| 2 | Age | Please specify your year of birth (B.E./A.D.): ............ |
| 3 | Highest level of education | 1. Primary education |
|  | completed | 2. Lower secondary education |
|  |  | 3. Upper secondary education or equivalent |
|  |  | 4. Diploma or equivalent |
|  |  | 5. Bachelor’s degree or higher |
|  |  | 6. No formal education |
| 4 | Occupation prior to incarceration (Please select only one option | 1. government officer / public sector employee /   state enterprise employee |
|  | that best describes your main occupation) | 2. private sector employee / company employee   / factory worker |
|  |  | 3. Sex worker |
|  |  | 4. Self-employed / Own business |
|  |  | 5. Farmer (e.g., crop farming, rice farming,   horticulture) |
|  |  | 6. General laborer (e.g., construction worker, daily  wage worker, motorcycle taxi driver) |
|  |  | 7. Unemployed |
|  |  | 8. Other (please specify)........................................ |
| 5 | Marital Status | 1. Single |
|  |  | 2. Married |
|  |  | 3. Divorced |
|  |  | 4. Widowed / Separated |
| 6 | Gender Identity | 1. Cisgender man / Cisgender woman |
|  | (Gender identity refers to an individual’s internal sense of gender, which may or may not correspond to sex assigned at birth.) | 2. Transgender woman |
|  |  | 3. Transgender man |
|  |  | 4. Other (please specify)........................................ |
|  |  |  |
|  |  |  |
| 7 | Sexual Orientation | 1. Heterosexual |
|  | (Sexual orientation refers to an individual’s pattern of emotional, romantic, or sexual attraction.) | 2. Homosexual |
|  |  | 3. Bisexual |
|  |  | 4. Other (please specify) ........................................ |
| 8 | In the past 1 year, have you  ever been diagnosed with | 1. No, I have never been diagnosed with an STIs   and have never undergone STIs testing. |
|  | or received treatment for a sexually transmitted infection (STIs)? | 2. Yes, I have been diagnosed with and/or received treatment for one or more STIs *(You may select more than one option)* |
|  |  | 2.1 Gonorrhea 2.2 Chlamydia |
|  |  | 2.3 Syphilis 2.4 Chancroid |
|  |  | 2.5 Lymphogranuloma venereum (LGV) |
|  |  | 2.6 Genital warts 2.7 Genital herpes |
|  |  | 2.8 Other (please specify)............................ |
|  |  | 3. Yes, I have experienced symptoms suggestive of an STIs but did not receive treatment or cannot recall the diagnosis. |

**Section 2: Behavioral Factors**

| **Item** | **Question** | **Response** |
| --- | --- | --- |
| 1 | In the past 12 months, have | 1. No |
|  | you had sexual intercourse? | 2. Yes |
| 2 | In the past 12 months, have | 1. No |
|  | you had vaginal sexual intercourse? | 2. Yes |
| 3 | In the past 12 months, have | 1. No |
|  | you had anal sexual intercourse? | 2. Yes |
| 4 | In the past 12 months, have | 1. No |
|  | you had oral sexual intercourse? | 2. Yes |
| 5 | In the past 12 months, have | 1. No |
|  | you ever bought or sold sexual | 2. Yes |
|  | services? |  |
| 6 | In the past 12 months, have | 1. No |
|  | you had sexual intercourse | 2. Yes |
|  | without using a condom? |  |
| 7 | In the past 12 months, have | 1. No |
|  | you used illicit drugs by | 2. Yes (please specify): ................................... |
|  | injection? |  |
| 8 | In the past 12 months, have | 1. No |
|  | you used non-injection illicit | 2. Yes (please specify): ................................... |
|  | drugs (e.g., oral, inhalation, or |  |
|  | smoking routes)? |  |
| 9 | In the past 12 months, have | 1. No |
|  | you used psychoactive | 2. Yes (please specify): ................................... |
|  | substances before or during |  |
|  | sexual intercourse? *(e.g.,* |  |
|  | *yaba/methamphetamine,* |  |
|  | *ice/crystal methamphetamine,* |  |
|  | *ketamine, alcohol)* |  |
| 10 | In the past 12 months, have | 1. No |
|  | you had tattoos or body | 2. Yes |
|  | piercings? *(e.g., tattooing,* |  |
|  | *piercing of the nose, ears,* |  |
|  | *or genital area)* |  |
| 11 | In the past 12 months, have | 1. No |
|  | you shared personal items | 2. Yes |
|  | with others? *(e.g., razors, nail* |  |
|  | *clippers, toothbrushes,* |  |
|  | *needles, towels)* |  |
