# Supplementary material for: Risk factors for syphilis among newly admitted prisoners: A case–control study
Source: PLoS One. 2026 Oct 16;21(10):e0360956. doi: 10.1371/journal.pone.0360956 (PMC13674709; doi:10.1371/journal.pone.0360956)
Supplement: Supplementary file 3 [file pone.0360956.s003.docx]

**Case Record Form**

**Title of the research project:** Factors associated with Syphilis infection among newly admitted inmates in a province of Thailand

**Code:** .................................................

**Instructions:** Please indicate your response by placing (✓) in the box that best reflects
your situation for each item.

| **Item** | **Question** | **Response** |
| --- | --- | --- |
| 1 | Syphilis infection status | 1. Reactive to syphilis screening test at prison admission |
|  |  | 2. Non-reactive to syphilis screening test at prison admission |
| 2 | HIV infection status | 1. Reactive to HIV screening test at prison admission  or previously diagnosed with HIV infection |
|  |  | 2. Non-reactive to HIV screening test at prison admission |
|  |  | 3. Declined HIV testing at prison admission |
|  |  | 4. Declined to disclose HIV infection status |

**Note:** All information recorded in this case record form will be kept strictly confidential
and used solely for the purposes of this study. Upon completion of the study, all paper-based case record forms will be destroyed using appropriate methods, specifically by shredding,
to prevent any unauthorized use of the data.
